# Severe Iron-Deficiency Anemia and Hypoalbuminemia Associated With Excessive Cow’s Milk Intake Driven by a Strong Belief in the Health Benefits of Cow’s Milk in a 16-Month-Old Girl: A Case Report

**DOI:** 10.7759/cureus.101657

**Published:** 2026-01-16

**Authors:** Tamotsu Gotou, Takahiro Hagihara, Yamato Wada, Kyoji Hashimoto, Futoshi Nagashima

**Affiliations:** 1 Department of Pediatric Emergency and Critical Care Medicine, Tottori Prefectural Central Hospital, Tottori, JPN; 2 Department of Emergency and Critical Care Medicine, Tottori Prefectural Central Hospital, Tottori, JPN; 3 Department of Emergency Medicine, Toyooka Hospital, Toyooka, JPN

**Keywords:** cow’s milk anemia, hypoalbuminemia, iron-deficiency anemia (ida), protein-losing enteropathy, red blood cell transfusion

## Abstract

Iron-deficiency anemia is common in infants and toddlers; however, excessive cow’s milk intake may lead to severe anemia and related complications. A 16-month-old girl presented with fever and progressive somnolence. She appeared unwell and had conjunctival pallor, pitting edema of the lower extremities, and a grade 4/6 systolic ejection murmur. Laboratory evaluation revealed severe microcytic hypochromic anemia (hemoglobin, 2.6 g/dL). Iron studies showed low serum iron (9 µg/dL) and ferritin (1.7 ng/mL) with a total iron-binding capacity of 286 µg/dL, consistent with iron deficiency. Serum albumin was markedly low (1.6 g/dL). The caregiver reported cow’s milk intake of approximately 1.0-1.5 L/day since early infancy. Chest radiography showed cardiomegaly (cardiothoracic ratio, 58%), and echocardiography demonstrated left ventricular dilation with preserved systolic function (ejection fraction, 62%). She was treated with leukoreduced packed red blood cell transfusion (10 mL/kg each on hospital days one and two), oral iron supplementation, and dietary counseling, including restriction of cow’s milk intake. Edema and overall clinical status improved, and she was discharged on hospital day seven. In infants and toddlers presenting with pallor, somnolence, and edema, cow’s milk intake should be quantified, and severe anemia and potential complications (e.g., hypoalbuminemia and thrombosis) should be promptly assessed.

## Introduction

Iron-deficiency anemia (IDA) is the most common nutritional anemia in children, and infants and young children are considered particularly susceptible to iron deficiency because rapid growth increases iron requirements [1]. Because iron deficiency during early childhood may exert long-term effects on neurodevelopment and cognitive function, early identification of reversible causes and timely intervention are clinically important [2].

In infants and young children, early introduction of unfortified cow’s milk and/or excessive cow’s milk intake can contribute to IDA through several mechanisms: (1) cow’s milk contains little iron; (2) calcium, casein, and other components inhibit absorption of non-heme iron; and (3) cow’s milk displaces iron-containing complementary foods (i.e., the perception that “milk is highly nutritious” may reduce intake of iron-rich solid foods) [1].

In low- and middle-income countries, where diets are more likely to be deficient in essential nutrients, animal-source foods, including milk, have been positioned as foods that promote children’s linear growth and weight gain, and milk has long been believed to play a unique role in growth and development [3]. However, reliance on unfortified cow’s milk as a primary nutrient source in early infancy warrants caution, as it may promote IDA via insufficient iron intake and reduced dietary diversity [3]. Indeed, a cross-sectional study of infants aged 0-12 months suggested that milk-based feeding may be associated with anemia, with a higher prevalence of IDA reported among infants primarily fed cow’s milk [4].

Severe IDA is also a risk factor for thrombosis, and pediatric case reports have described cerebral venous sinus thrombosis (CVST) occurring in the setting of cow’s milk overconsumption-associated IDA [5].

For children aged one to five years, cow’s milk intake is commonly recommended to be limited to approximately ≤500 mL/day; intake substantially above this is generally considered excessive and may increase the risk of IDA [6]. Although cow’s milk-associated severe IDA has been increasingly recognized globally, published reports from Japan remain limited [7].

In the present case, based on the clinical course of severe IDA in the context of excessive cow’s milk intake and selective eating in early childhood, we describe key elements of the diagnostic process and therapeutic intervention and discuss the clinical implications of cow’s milk-associated anemia, which is considered rare in Japan.

## Case presentation

A 16-month-old girl (weight, 9.0 kg), whose weight-for-age z score was −0.9 (SDS) based on the Japanese growth reference [8] and within the normal range (z ≥ −2) according to the WHO Child Growth Standards [9], presented to the emergency department with fever and progressive somnolence. According to the caregiver, somnolence had gradually worsened over the preceding two weeks, and the child spent increasing amounts of time lying down. Two days before the presentation, she visited an outpatient clinic for fever and was noted to be pale; however, further evaluation was not performed because the caregiver reported that pallor was her usual condition. Fever persisted and somnolence progressed, prompting presentation to our emergency department.

There was no relevant past medical history and no family history of hematologic disease. The caregiver was a single parent from Southeast Asia. The patient was born to a mother of Southeast Asian origin and a Japanese father. The neonatal nursing record documented that the caregiver informed a midwife of her intention to feed cow’s milk immediately after birth. Cow’s milk was introduced shortly after discharge from the maternity unit, and the daily intake gradually increased thereafter. No other unusual dietary practices, including pica, were reported or elicited aside from the excessive cow’s milk consumption. After the child turned one year old, the mother returned to work, and the child spent more time with her older sister (three years older); cow’s milk intake increased further, reaching approximately 1.0-1.5 L/day immediately before presentation.

On arrival, the patient was in poor general condition and was unable to sit independently. Vital signs were as follows: heart rate, 198 beats/minute; respiratory rate, 44 breaths/minute; blood pressure, 132/52 mmHg; and oxygen saturation, 98% on room air. She had conjunctival pallor, periorbital (eyelid) edema, and pitting edema of the lower extremities, and a grade 4/6 systolic ejection murmur was audible. There was no hepatosplenomegaly. Work of breathing was increased, and no obvious traumatic marks were observed.

Laboratory evaluation revealed severe microcytic hypochromic anemia (hemoglobin, 2.6 g/dL), and red blood cell morphology was unremarkable. Iron studies supported iron deficiency (serum iron, 9 µg/dL; ferritin, 1.7 ng/mL; total iron-binding capacity, 286 µg/dL), and serum albumin was 1.6 g/dL, indicating hypoalbuminemia. Fecal occult blood testing was negative on a single sample; repeat testing was not performed because there were no gastrointestinal symptoms or signs suggestive of ongoing bleeding, and the anemia improved promptly after nutritional intervention. The direct and indirect Coombs tests were negative. The platelet count was 34.4 × 10^4^/µL, and thrombocytosis was not present. Specific immunoglobulin E (IgE) tests for cow’s milk and casein were positive (Table 1). The hemoglobin level (2.6 g/dL) met the WHO definition of severe anemia in children aged 6-59 months (hemoglobin <7.0 g/dL) [10].

**Table 1:** Laboratory data. On admission, laboratory tests showed severe microcytic hypochromic anemia and marked hypoalbuminemia, consistent with an iron-deficiency pattern. RBC = red blood cells; Hb = hemoglobin; Hct = hematocrit; MCV = mean corpuscular volume; MCH = mean corpuscular hemoglobin; MCHC = mean corpuscular hemoglobin concentration; TP = total protein; Alb = albumin; T-Bil = total bilirubin; AST = aspartate aminotransferase; ALT = alanine aminotransferase; LDH = lactate dehydrogenase; Fe = serum iron; UIBC = unsaturated iron-binding capacity; TIBC = total iron-binding capacity

Category	Parameter	Result	Reference range
Hematology	RBC	249 × 10^4^/µL	380–480
	Hb	2.6 g/dL	11.0–15.0
	Hct	11.2%	34.0–48.0
	MCV	45 fL	83.0–99.0
	MCH	10.4 pg	28.4–34.6
	MCHC	23.2 %	32.5–35.5
	Reticulocyte count (absolute)	5.9 × 10^4^/µL	8.0–22.0
	Platelet count	34.4 × 10^4^/µL	12.0–35.0
Immunohematology	Direct/Indirect Coombs test	Negative/Negative	Negative/Negative
Chemistry	TP	3.5 g/dL	6.7–8.0
	Alb	1.6 g/dL	3.4–4.9
	T-Bil	0.2 mg/dL	0.0–1.0
	AST	53 IU/L	8–30
	ALT	18 IU/L	5–35
	LDH	318 IU/L	106–211
Iron studies	Fe	9 µg/dL	55–180
	UIBC	277 µg/dL	130–320
	TIBC	286 µg/dL	317–395
	Ferritin	1.71 ng/mL	6.23–138
Other	Fecal occult blood	Negative	Negative

**Figure 1:**
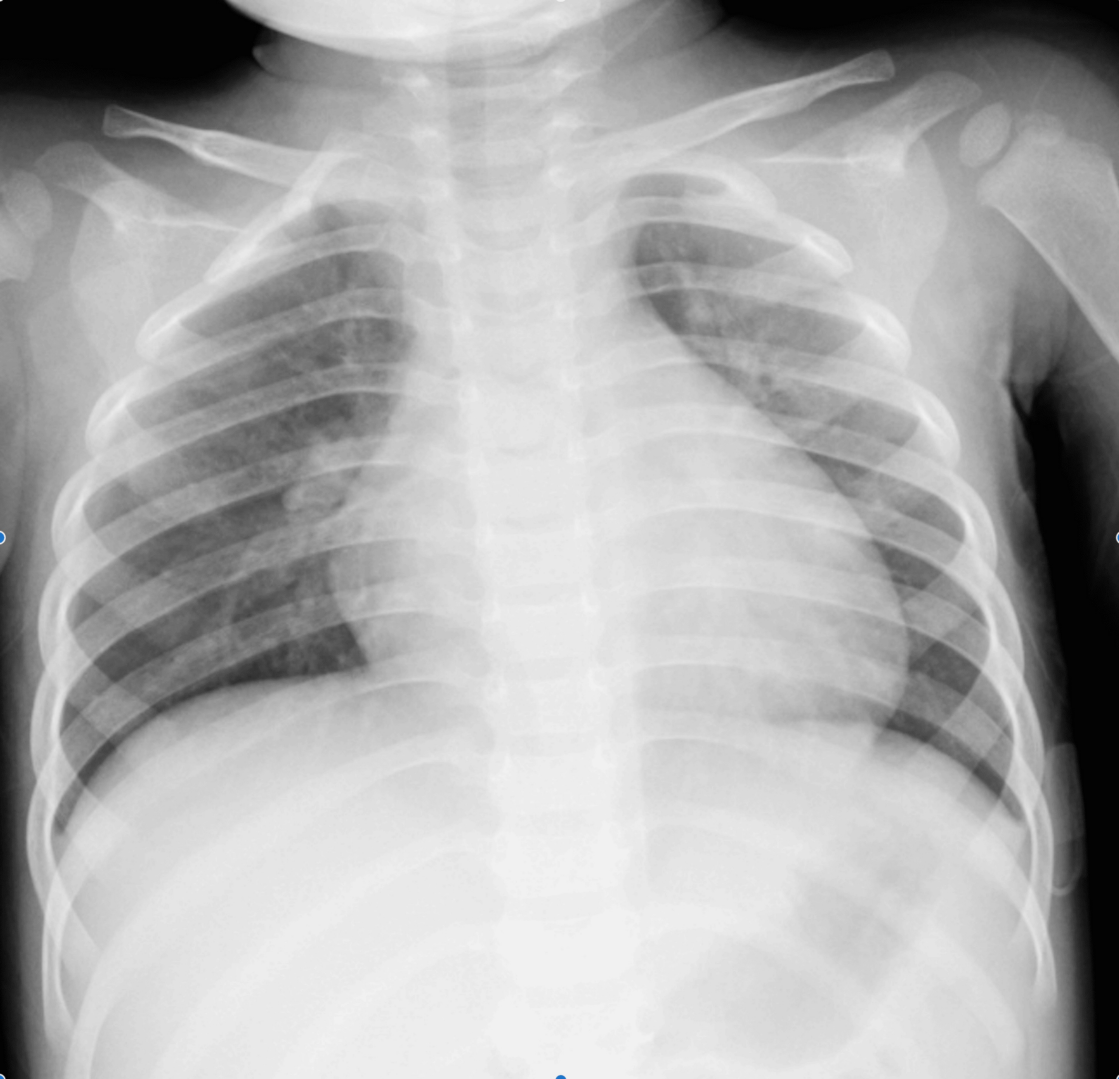
Chest radiograph on admission (cardiomegaly). Cardiomegaly with a cardiothoracic ratio of 58% was observed on the chest radiograph at admission.

Chest radiography showed cardiomegaly (cardiothoracic ratio, 58%) (Figure 1).

Echocardiography demonstrated left ventricular dilation (left ventricular end-diastolic diameter, 34 mm) with preserved systolic function (ejection fraction, 62%). There was no mitral or aortic regurgitation, and tricuspid regurgitation was minimal. The patient was diagnosed with severe IDA associated with excessive cow’s milk intake, complicated by hypoalbuminemia with sensitization to cow’s milk protein. Her clinical and anthropometric findings were not consistent with marasmus, and she had no evidence of failure to thrive. Neurodevelopmental assessment revealed age-appropriate development without apparent delay.

**Figure 2:**
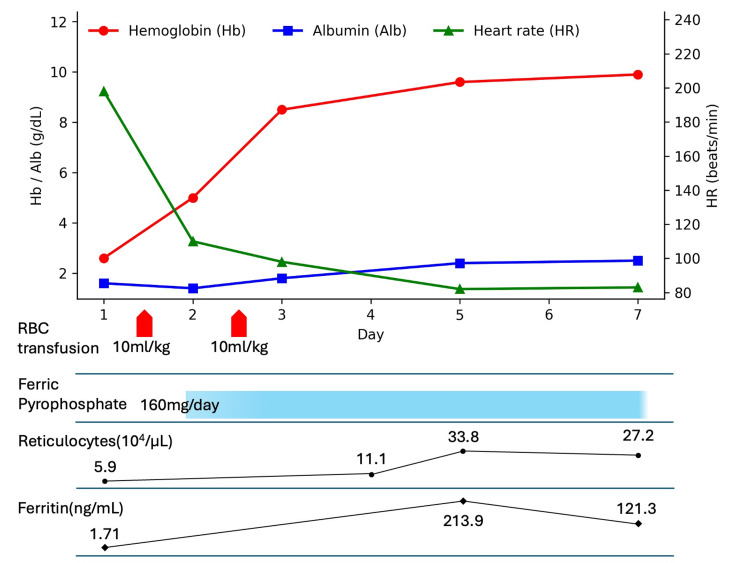
Clinical course. Temporal changes in hemoglobin (Hb), albumin (Alb), and heart rate (HR) during hospitalization. The timing and doses are shown for leukoreduced packed red blood cell (RBC) transfusion (10 mL/kg each on the day of admission and on hospital day two) and initiation of oral iron supplementation (ferric pyrophosphate, 160 mg/day). Absolute reticulocyte count (×10^4^/µL) and ferritin (ng/mL) are also shown. The patient was discharged on hospital day seven.

Given the extremely low hemoglobin level (2.6 g/dL) with clinical features suggesting impaired oxygen delivery (tachycardia and lethargy), leukoreduced packed red blood cells were transfused at 10 mL/kg on the day of admission and on hospital day two. Hemoglobin rose from 2.6 g/dL to 8.5 g/dL by hospital day three and to 9.9 g/dL by hospital day seven, without further transfusion. Oral iron supplementation (ferric pyrophosphate, 160 mg/day) was started on hospital day two, corresponding to approximately 2 mg/kg/day of elemental iron, in accordance with the dosing recommendations in the manufacturer’s prescribing information. Ferritin increased from 1.7 ng/mL to 213.9 ng/mL by hospital day five. The absolute reticulocyte count rose from 5.9 × 10^4^/µL to 33.8 × 10^4^/µL by hospital day five; notably, the baseline reticulocyte count was relatively low for the severity of anemia, consistent with iron-restricted erythropoiesis, and the subsequent reticulocytosis indicated hematologic recovery after treatment. By hospital day two, she was able to sit independently and take oral intake, and pitting edema of the eyelids and pretibial region resolved during hospitalization. Tachycardia improved promptly after transfusion, and vital signs remained stable thereafter; follow-up echocardiography demonstrated preserved systolic function with resolution of the previously observed left ventricular dilatation. A dietitian provided dietary counseling to correct cow’s milk intake, and, with the mother’s consent, the hospital social worker informed the community public health nurse to arrange post-discharge support. She was discharged on hospital day seven (Figure 2).

After discharge, oral iron was continued for three months, and no recurrence of anemia was confirmed; follow-up ended at six months. Psychomotor development remained age-appropriate throughout the follow-up period, with no apparent delay.

## Discussion

In this case, excessive cow’s milk intake was associated with severe IDA and hypoalbuminemia. Excessive cow’s milk intake can promote iron deficiency through multiple mechanisms. First, it displaces iron-containing foods. Cow’s milk contains little iron, and cow’s milk alone cannot meet the iron requirements of infants and toddlers [1]. Second, cow’s milk is rich in calcium and casein, which may reduce the bioavailability of dietary non-heme iron through physicochemical binding and interference with intestinal iron uptake, thereby decreasing net iron absorption [1,11].

In addition to dietary displacement, excessive cow’s milk intake in infants and toddlers may increase gastrointestinal microbleeding [1,11]. Therefore, evaluation of IDA should include a quantitative dietary history and, when clinically indicated, assessment for gastrointestinal blood loss, including fecal occult blood testing [11,12]. In this case, fecal occult blood testing was negative; however, a single test may yield a false-negative result, and repeat assessment may be considered depending on the clinical context.

Severe IDA associated with excessive cow’s milk intake has been reported in conjunction with protein-losing enteropathy (PLE) [13,14]. Proposed mechanisms include cow’s milk-induced enteropathy and immune-mediated intestinal injury [13]. In our patient, serum cow’s milk- and casein-specific IgE were detectable, consistent with sensitization; however, the overall clinical course was not indicative of clinically significant IgE-mediated cow’s milk allergy. Allergic manifestations were limited, and the patient improved following iron supplementation and dietary modification, including reduction of excessive cow’s milk intake and reintroduction of age-appropriate complementary foods. Accordingly, we interpreted the IgE findings as incidental and attributed the presentation primarily to the nutritional consequences of excessive cow’s milk intake. The favorable response to these interventions supports a substantial contribution from reversible, diet-related factors.

Severe IDA may also be accompanied by a broad range of systemic manifestations. Early clinical features include pallor, fatigue/lethargy, irritability, poor feeding or decreased appetite, and reduced activity tolerance; physical examination may reveal tachycardia and a functional systolic murmur [11]. When IDA is profound or prolonged, it may be associated with pica, growth faltering, and neurobehavioral or developmental concerns [2,11], and, in severe cases, may contribute to high-output cardiac strain or heart failure [11]. In addition, reactive thrombocytosis can occur in severe IDA, and pediatric CVST has been reported in the setting of IDA related to excessive cow’s milk intake [5]. In the present case, the platelet count was within the reference range (34.4 × 10^4^/µL), and thrombocytosis was not observed. Accordingly, in patients with severe IDA and/or reactive thrombocytosis, clinicians should assess for neurological symptoms (e.g., headache, seizures, altered mental status) and consider neuroimaging when clinically indicated [5].

The cornerstone of treatment is removal of the cause (restriction of cow’s milk intake, introduction of solid foods, and promotion of iron-containing foods) together with iron supplementation [1,11,12]. For therapeutic supplementation, oral elemental iron is typically administered at 3-6 mg/kg/day (as elemental iron) [11]. For prophylactic supplementation in at-risk infants and toddlers, recommended dosing includes 1 mg/kg/day (e.g., exclusively breastfed infants after four months until iron-rich complementary foods are established) and 2 mg/kg/day (e.g., preterm/low-birth-weight infants from 1 month to 12 months) [11]. After initiating oral iron, reticulocytosis is generally observed within 7-10 days, and hemoglobin begins to rise within 2-4 weeks [11]. Because repletion of iron stores lags behind hemoglobin normalization, continuation for at least approximately three months is recommended [11,12]. Clinical follow-up with repeat complete blood count (± reticulocyte count) within 2-4 weeks is useful to confirm an appropriate hematologic response, with continued monitoring thereafter as clinically indicated. In the present case, oral iron was continued for three months, and follow-up through six months confirmed no recurrence of anemia.

In severe cases with circulatory or respiratory compromise, transfusion may be required. Intravenous iron can be considered when oral iron is not tolerated, absorption is impaired, or the response is inadequate [12]. In this case, the response to treatment was favorable, and the patient became able to tolerate oral intake; thus, intravenous iron was unnecessary. Treatment should be individualized based on symptom severity and the feasibility of follow-up.

Excessive cow’s milk intake can occur when caregivers perceive cow’s milk as “nutritious” or “good for growth.” Therefore, early identification through dietary counseling and routine child health visits is important [1,11]. In general, for children aged one to five years, cow’s milk intake is recommended to be limited to approximately ≤500 mL/day, prioritizing a varied diet that includes iron-containing solid foods [1].

The caregiver in this case was a single parent from Southeast Asia, which may indicate limited access to in-home childcare support. Limited social support can contribute to the delayed introduction of complementary foods and increased reliance on cow’s milk; therefore, dietary history-taking should include quantification of cow’s milk intake and consideration of the family’s social background. Clinicians may also need to clarify that cow’s milk is a supplemental beverage rather than a substitute for a balanced diet and provide practical examples of iron-containing complementary foods.

Caregivers’ perceptions that cow’s milk is intrinsically “healthy” or “growth-promoting” may be shaped by commercial promotion. The impact of marketing of breast-milk substitutes on feeding practices has been discussed [15], and WHO/UNICEF materials note that formula marketing and cross-promotion can influence caregivers’ decisions [16,17]. Surveys in low- and middle-income countries have described follow-up formulas and growing-up milks with labeling similar to infant formula, potentially facilitating cross-promotion [18], and an analysis in East Asia and the Pacific reported correlations between implementation of the International Code of Marketing of Breast-milk Substitutes, maternity protection, and consumption of commercial milk products [19]. While animal-source foods, including cow’s milk, can contribute to child nutrition, excessive intake that displaces iron-containing complementary foods should be avoided [3]. In this case, the mother’s early intention to introduce cow’s milk immediately after birth, together with limited external support in a household with a foreign-born caregiver, suggests that social and informational factors may have contributed; thus, when cow’s milk-associated anemia is identified, assessment should encompass both dietary intake and the need for social support.

Reports from Japan are limited [7], and the perceived rarity of cow’s milk-associated severe IDA may be partly explained by national guidance on breastfeeding and weaning, which generally recommends introducing cow’s milk as a beverage after one year of age while emphasizing age-appropriate complementary foods [20]. The perceived rarity may also reflect under-ascertainment and publication bias, as clinically managed cases may go unreported and Japanese-language reports may be less visible internationally. Notwithstanding this perceived rarity, serious complications such as PLE and thrombosis have been reported [5,13,14]. Therefore, in infants and toddlers presenting with severe anemia, quantifying cow’s milk intake, assessing complementary foods, and initiating early nutritional intervention in parallel with the diagnostic evaluation may be clinically useful.

## Conclusions

This case suggests that excessive intake of unfortified cow’s milk may contribute to severe IDA and hypoalbuminemia, potentially by displacing iron-containing complementary foods. Caregivers’ beliefs that cow’s milk is inherently “healthy” or “growth-promoting,” which may be reinforced by social circumstances and commercial messaging, may encourage overreliance on milk, particularly in households with limited resources or support. When cow’s milk-associated IDA is suspected, clinicians may benefit from quantifying daily intake, addressing misconceptions through practical and culturally sensitive dietary counseling, and considering the need for social support to facilitate sustained dietary change and reduce the likelihood of recurrence.
